# Bleeding complications in patients with gastrointestinal cancer and atrial fibrillation treated with oral anticoagulants

**DOI:** 10.1002/cam4.4012

**Published:** 2021-06-11

**Authors:** Anne Gulbech Ording, Mette Søgaard, Flemming Skjøth, Erik Lerkevang Grove, Gregory Y. H. Lip, Torben Bjerregaard Larsen, Peter Brønnum Nielsen

**Affiliations:** ^1^ Unit for Thrombosis and Drug Research Department of Cardiology Aalborg University Hospital Aalborg Denmark; ^2^ Aalborg Thrombosis Research Unit Aalborg University Aalborg Denmark; ^3^ Unit of Clinical Biostatistics Aalborg University Hospital Aalborg Denmark; ^4^ Department of Cardiology Aarhus University Hospital Aarhus N Denmark; ^5^ Department of Clinical Medicine Faculty of Health Aarhus University Aarhus C Denmark; ^6^ Liverpool Centre for Cardiovascular Sciences University Liverpool and Liverpool Heart & Chest Hospital Liverpool UK

**Keywords:** atrial fibrillation, gastrointestinal neoplasms, hemorrhage, anticoagulants, factor Xa inhibitors

## Abstract

**Background:**

Direct oral anticoagulants (DOACs) may increase the risk of gastrointestinal (GI) bleeding in patients with atrial fibrillation (AF) and GI cancer compared with vitamin K antagonists (VKA).

**Methods:**

We conducted a Danish nationwide cohort study comparing the bleeding risk associated with DOAC versus VKA in patients with AF and GI cancer. We calculated crude bleeding rates per 100 person‐years (PYs) for GI and major bleeding. We then compared rates of bleeding at 1 year after initial oral anticoagulation filled prescription by treatment regimen using inverse probability of treatment weighting and Cox regression.

**Results:**

The unweighted study population included 1476 AF patients with GI cancer (41.6% women, median age 78 years) initiating a DOAC and 652 initiating a VKA. One‐year risk of GI bleeding was 5.0% in the DOAC group and 4.7% in the VKA group with a corresponding weighted hazard ratio (HR) of 0.95 (95% confidence interval [CI]: 0.63, 1.45). For patients with active cancer, weighted GI bleeding rates were slightly higher in both the VKA and DOAC group, and the weighted HR was 1.00 (95% CI: 0.53, 1.88). The HR was 1.12 (95% CI: 0.71, 1.76) for all bleedings. Hazard ratios for GI bleeding were 0.61 (95% CI: 0.25, 1.52) for patients with upper GI cancer, and 0.92 (95% CI: 0.58, 1.46) in patients with colorectal cancer.

**Conclusion:**

Evidence from this nationwide cohort study suggests a comparable 1‐year risk of bleeding associated with DOAC compared with VKA among patients with AF and GI cancer.

## Graphical abstract

One‐year risk of gastrointestinal bleeding was 5.0% in the DOAC group and 4.7% in the VKA group. The hazard ratio comparing DOAC with VKA for GI bleeding was 0.95 (0.63, 1.45).

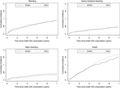

## 1 INTRODUCTION

Atrial fibrillation (AF) affects more than 44 million individuals worldwide,^1^ and patients with AF are at five‐fold increased risk of stroke compared with individuals without AF.^1^ Similar to AF, cancer incidence rises with age, and because many cancer types and their treatments interact with the coagulation system, cancer patients are at increased risk of cardiovascular events.^2^ Malignancy concurrent with AF is an important clinical challenge because of increased bleeding and thrombotic risk.^3^

Vitamin K antagonist (VKA) oral anticoagulants (OAC) have been used for stroke prevention in AF, but the direct oral anticoagulants (DOACs) rivaroxaban, dabigatran, apixaban, and edoxaban are now recommended over VKA in international guidelines.^4^, ^5^, ^6^ DOACs are increasingly prescribed to AF patients with cancer, though clinical trials investigating the efficacy and safety of DOACs included only few, mainly low‐risk, cancer patients.^7^, ^8^, ^9^, ^10^ DOAC may act as topical anticoagulants and predispose to bleeding in patients with gastrointestinal (GI) cancers, particularly upper GI cancers.^11^, ^12^ Posthoc analyses from clinical trials comparing DOACs with VKA for stroke prevention in AF showed similar bleeding risk in patients with a history of cancer.^10^, ^13^, ^14^ Cohort studies have also shown similar bleeding risk in patients treated with VKA versus DOACs.^15^, ^16^, ^17^ These studies; however, pooled patients across cancer sites, although the bleeding risk may differ by cancer type.

We used Danish nationwide registries, to compare bleeding risk associated with DOAC versus VKA in patients with AF and history of GI cancer.

## 2 MATERIALS AND METHODS

### 2.1 Setting and data sources

The Danish National Health Service provides tax‐supported health care to all residents.^18^ Nationwide registries track vital status, diagnoses, and procedures for the entire population. Data is linked across registries using the unique civil registration number assigned to all Danish residents at birth or upon immigration. Migration, sex, and vital status are tracked by the Civil Registration System (CRS).^19^ The Danish National Patient Registry (DNPR) covering all Danish hospitals has recorded all clinical inpatient discharge diagnoses since 1977 and diagnoses made at outpatient clinic visits since 1995.^20^ The Danish National Prescription Database (DNPD) records information on prescription claims from outpatient pharmacies since 2004 using the Anatomical Therapeutic Chemical (ATC) Classification System.^21^ The Danish Cancer Registry (DCR) records all incident cancer cases in Denmark since 1943, with mandatory recording since 1987, including information on morphology, histology, and stage at diagnosis.^22^

### 2.2 Design and study population

We used the DNPR to identify a cohort of nonvalvular AF patients with inpatient or outpatient hospital‐based diagnoses who initiated a DOAC or a VKA between 1 August 2011 and 30 June 2018. We restricted the cohort to OAC naïve patients, defined as no prior experience of OAC, who had a diagnosis of GI cancer in the DCR before first OAC prescription claim (*N* = 2619; index date).^21^ We excluded patients with other indications for OAC, e.g., prevalent diagnosis of venous thromboembolism (*N* = 181) and valve disease (*N* = 301).

We obtained information from the DNPR on inpatient and outpatient diagnoses of comorbidities at index date including cardiovascular and metabolic diseases. Use of cardiovascular medication within 90 days before index was collected from the DNPD. Cancer‐targeted treatment recorded within six months before index date included GI surgery, chemotherapy, and radiotherapy. Active cancer was defined as a diagnosis of GI cancer within the previous six months, a diagnosis of metastasis or receipt of chemotherapy or radiotherapy within the previous six months. We combined covariate information into modified HAS‐BLED scores (the L component of labile INR values was not included) as a measure of baseline bleeding risk (Supporting Information Table S1 provide codes and definitions).

### 2.3 Bleeding endpoints and follow‐up

Bleeding events were examined as any episode of GI bleeding recorded at a hospital contact, major bleeding outside the GI system, intracranial hemorrhage, and as a composite of all bleedings recorded in the DNPR after index. Patients were followed from index to 1 year to ascertain the first clinically relevant bleeding event regardless of extent and severity, with censoring at emigration, death or 31 December 2018, whichever came first.

### 2.4 Statistical analyses

Descriptive summaries of baseline characteristics stratified by first‐time DOAC or VKA prescription claim were presented as proportions for discrete variables and as medians (interquartile range [IQR]) for continuous variables.

Analyses were conducted under the intention to treat principle assuming continuous treatment during study follow‐up based on the initial treatment allocation. To account for baseline confounding, we used an inverse probability of treatment weighting (IPTW) approach to obtain estimates representing population average treatment effects on pseudo‐cohorts of patients treated with VKA or DOACs with comparable characteristics. The weights were derived using generalized boosted models based on up to 10,000 regression trees.^23^ The underlying regression trees included information on age, sex, cancer type (as shown in Table 1), cancer stage (as shown in Table 1), and dichotomous variables including history of stroke, diabetes, hypertension, heart failure, bleeding, and use of lipid‐lowering drugs. The balance between treatment‐populations was evaluated by standardized differences of all measured baseline covariates, using a threshold of 0.1 to indicate imbalance.^24^

**TABLE 1:** Characteristics of patients with atrial fibrillation and a history of gastrointestinal cancer according to initial prescription claim for a DOAC or VKA

Characteristic	Unweighted population			Weighted population
	DOAC group	VKA group	Standardized difference	Standardized difference
Participants	1476	652		
Females	44.4 (655)	35.4 (231)	0.1835	0.0045
Median age, y	78.5 (72.0–85.0)	78.0 (72.0–84.0)	0.1071	0.0011
Cancer type^a^				
Esophagus	2.8 (41)	3.8 (25)	0.0591	0.0049
Stomach	5.1 (75)	4.8 (31)	0.0151	0.0118
Small intestine	1.2 (17)	1.2 (8)	0.0069	0.0099
Colorectal	86.5 (1277)	84.7 (552)	0.0528	0.0063
Pancreas	1.8 (27)	1.4 (9)	0.0357	0.0035
Liver	2.0 (30)	3.1 (20)	0.0657	0.0036
Anal canal	1.2 (17)	1.7 (11)	0.0453	0.0021
Metastasis^b^	2.2 (32)	3.8 (25)	0.0978	0.0926
Active cancer	40.0 (591)	50.6 (330)	0.2136	0.2150
Cancer treatment^b^				
Chemotherapy	8.1 (120)	10.7 (70)	0.0893	0.0750
Radiation therapy	30.3 (447)	41.3 (269)	0.2304	0.2319
Surgery	21.4 (316)	29.0 (189)	0.1752	0.1661
Cancer stage				
Localized	49.5 (730)	50.6 (330)	0.0231	0.0249
Regional	24.7 (364)	25.9 (169)	0.0290	0.0018
Distant	4.8 (71)	6.7 (44)	0.0831	0.0721
Missing/unknown	21.1 (311)	16.7 (109)	0.1114	0.0089
Comorbidities				
Heart failure	23.3 (344)	29.6 (193)	0.1431	0.0081
Diabetes	17.1 (253)	18.4 (120)	0.0331	0.0051
Hypertension	57.7 (851)	62.7 (409)	0.1038	0.0115
Stroke	14.6 (215)	9.5 (62)	0.1559	0.0208
Systemic embolism	0.7 (10)	0.8 (5)	0.0106	0.0418
Myocardial infarction	10.0 (147)	11.5 (75)	0.0499	0.0092
Ischemic heart disease	23.7 (350)	27.5 (179)	0.0858	0.0523
Cardiomyopathy	1.8 (26)	2.1 (14)	0.0279	0.0132
Obesity	8.5 (125)	8.6 (56)	0.0043	0.0285
Hyperthyroidism	3.8 (56)	3.7 (24)	0.0060	0.0337
Chronic pulmonary disease	15.3 (226)	15.5 (101)	0.0050	0.0157
Liver disease	0.3 (5)	‐ (<5)	0.0399	0.0143
Renal disease	6.0 (88)	11.3 (74)	0.1925	0.1730
Previous bleeding	24.1 (355)	23.8 (155)	0.0065	0.0099
HAS‐BLED score				
0	1.0 (15)	1.7 (11)	0.0581	0.0428
1–2	48.0 (708)	42.3 (276)	0.1134	0.1048
3+	51.0 (753)	56.0 (365)	0.0997	0.0947
CHA_2_DS_2_‐VASc score				
0	1.0 (15)	2.1 (14)	0.0907	0.0592
1	7.0 (103)	7.1 (46)	0.0030	0.0334
2–4	64.2 (411)	65.2 (425)	0.0214	0.0000
5+	27.8 (411)	25.6 (167)	0.0505	0.0032
Medication				
Warfarin		100 (652)		
Apixaban	40.9 (603)	—	—	—
Dabigatran	22.4 (331)	—	—	—
Edoxaban	0.9 (14)	—	—	—
Rivaroxaban	35.8 (528)	—	—	—
DOAC standard dose	58.7 (867)	—	—	—
DOAC reduced dose	41.3 (609)	—	—	—
Renin‐angiotensin inhibitor (ACE/ARB)	36.2 (535)	35.1 (229)	0.0235	0.0927
Calcium channel blockers	20.4 (301)	25.8 (168)	0.1278	0.1078
Beta blockers	61.8 (912)	62.9 (410)	0.0226	0.0252
Diuretics	34.5 (509)	43.7 (285)	0.1899	0.1426
Digoxin	23.6 (349)	24.4 (159)	0.0174	0.0170
Lipid lowering drugs	28.3 (418)	27.3 (178)	0.0227	0.0036
Aspirin	25.1 (371)	30.2 (197)	0.1137	0.1034
Non‐steroidal anti‐inflammatory drugs	6.9 (102)	6.0 (39)	0.0378	0.0463
Amiodarone	3.4 (50)	4.6 (30)	0.0620	0.0527
Clopidogrel, ticagrelor, prasugrel	9.7 (143)	8.9 (58)	0.0273	0.0147

Note

Data are the median (interquartile range) or the % (number of patients), as indicated; counts were suppressed for observations with less than five incidents, to prevent disclosure of potentially identifiable information.

Abrreviations: DOAC, direct oral anticoagulant; VKA, vitamin K antagonist.

^a^ Cancer types are not mutually exclusive.

^b^ Recorded within 6 months before index.

We used the Aalen‐Johansen estimator to compute weighted cumulative incidence curves of bleeding accounting for competing risk of death.^25^ Mortality risk was also depicted as a lone outcome by means of Kaplan‐Meier estimates. We calculated bleeding rates per 100 person‐years (PYs) within the unweighted and weighted treatment populations. To estimate the relative risk of bleeding for DOAC versus VKA users (reference) we calculated cause‐specific hazard ratios using weighted Cox proportional hazards regression. In a sensitivity analysis, we excluded patients initiating reduced‐dose DOAC, which may indicate a perceived high baseline bleeding risk. Supplementary analyses were done specifically for patients with active cancer and for patients with upper GI cancer (defined as cancer in the esophagus, stomach, small intestine, pancreas, and liver including the bile duct and gall bladder) or colorectal cancer. Due to few events in these subgroups, we only presented results for GI bleeding and the composite of all bleedings. All weights were recalculated for the additional analyses as the study population was changed. Point estimates were reported with 95% confidence intervals (CI).

Analyses were conducted using SAS software version 9.4 (SAS Institute) and Stata MP version 16 (StataCorp.).

## 3 RESULTS

The study included 2128 patients with AF (41.6% women, median age 78 years) and a history of GI cancer who claimed a prescription for DOAC (*N* = 1476) or VKA (*N* = 652). Apixaban was the most commonly prescribed DOACs (40.9%) followed by rivaroxaban (35.8%), dabigatran (22.4%), and edoxaban (0.9%). All patients in the VKA group received warfarin. Colorectal cancer accounted for 86% of cancers and 43% of the cohort had active cancer. Median time from primary GI cancer diagnosis to OAC prescription was 1948 days (IQR: 587, 4090) in the DOAC group and 1537 days (IQR: 270, 3810 days) in the VKA group, whereas the median number of days between AF diagnosis and OAC prescription was 15 days (IQR: 4, 332 days) in the DOAC group and 25 days (IQR: 7, 310 days) in the VKA group.

VKA patients more often had comorbid heart failure (29.6% vs. 23.3%), hypertension (62.7% vs. 57.7%), ischemic heart disease (27.5% vs. 23.7%), and renal disease (11.3% vs. 6.0%) and more dispensed prescriptions for most cardiovascular drugs (Table 1). More patients received standard‐dose DOAC (59%) than reduced‐dose (41%). Patients treated with a reduced‐dose were older (median age 84 vs. 75 years), more often female (55.8% vs. 36.3%), rarely had active cancer but a higher HAS‐BLED score, than patients treated with standard‐dose DOAC (Supporting Information Table S2).

After propensity score weighting, the treatment groups were generally well‐balanced across all measured covariates. Visual inspection of the propensity score distribution showed good overlap between treatment groups, with no sign of violation of the positivity assumption with scores approaching zero (Supporting Information Figure S1). The proportions of patients with active cancer differed between treatment groups with more patients with active cancer in the VKA group.

### 3.1 Bleeding in DOAC versus VKA

**FIGURE 1:**
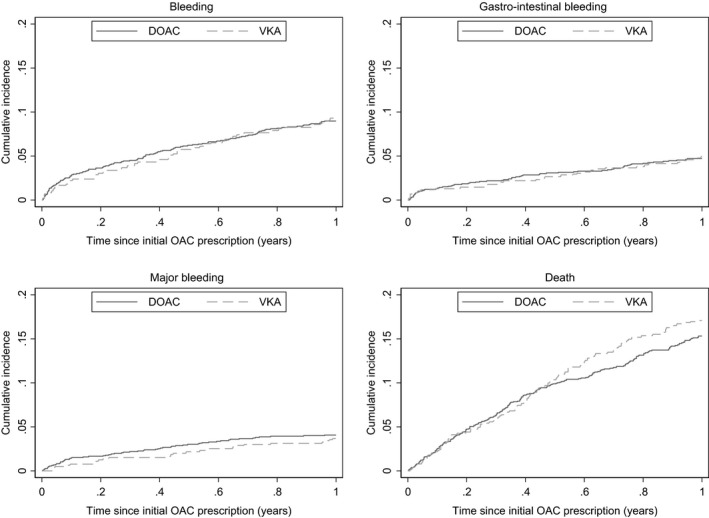
Weighted 1‐year bleeding and mortality risk in patients with atrial fibrillation and a history of gastrointestinal cancer

There were 128 bleeding events in DOAC users and 57 in VKA users with corresponding unweighted bleeding rates of 10.36 and 10.17, respectively. In both treatment groups, we observed an initially relatively steep bleeding curve, which levelled off over time (Figure 1).

Weighted 1‐year GI bleeding rates were comparable for DOAC (*N* = 66) and VKA (*N* = 31) users with 5.36 per 100 PYs for DOAC and 5.62 for VKA and a weighted HR of 0.95 (95% CI: 0.63, 1.45). Similar results were observed for the combined bleeding endpoint, HR = 0.95 (95% CI: 0.70, 1.29). Intracranial bleeding was rare with 7 cases in each group, but a HR = 0.32 (95% CI: 0.12, 0.86). In analyses of standard‐dose DOAC, the hazard increased slightly for all bleeding endpoints, except for intracranial bleeding (Table 2).

**TABLE 2:** One‐year rates and HRs for bleeding events in patients with atrial fibrillation and a history of gastrointestinal cancer according to initial prescription claim for a DOAC or VKA

Outcome	Group	Bleeding events, *n*	Unweighted rate per 100 person‐years (95% CI)	Weighted rate per 100 person‐years (95% CI)	Weighted HR (95% CI)	Standard DOAC dose Weighted HR (95% CI)
Any bleeding	DOAC	128	10.36 (8.72, 12.33)	10.53 (8.85, 12.62)	0.95 (0.70, 1.29)	1.11 (0.79, 1.56)
	VKA	57	10.17 (7.84, 13.18)	11.02 (8.47, 14.58)	Ref	Ref
Major bleeding	DOAC	59	4.65 (3.60, 6.00)	4.68 (3.63, 6.12)	1.11 (0.69, 1.79)	1.21 (0.72, 2.03)
	VKA	23	4.01 (2.66, 6.03)	4.17 (2.77, 6.58)	Ref	Ref
GI bleeding	DOAC	66	5.21 (4.09, 6.63)	5.36 (4.22, 6.90)	0.95 (0.63, 1.45)	1.18 (0.73, 1.89)
	VKA	31	5.41 (3.81, 7.70)	5.62 (3.95, 8.26)	Ref	Ref
Intracranial bleeding	DOAC	7	0.54 (0.26, 1.13)	0.50 (0.24, 1.20)	0.32 (0.12, 0.86)	0.23 (0.06, 0.96)
	VKA	7	1.20 (0.57, 2.52)	1.57 (0.75, 3.86)	Ref	Ref

Abbreviations: DOAC, direct oral anticoagulant; VKA, vitamin K antagonist.

For patients with active cancer, the weighted GI bleeding rates were similar in both OAC groups (27 and 14 bleeding events, respectively, in DOAC and VKA patients), weighted HR = 1.00 (95% CI: 0.53, 1.88). For the composite of all bleedings, a consistently higher rate was observed among DOAC users with 55 versus 25 bleeding events in the VKA group and a HR = 1.12 (95% CI: 0.71, 1.76). In the subgroups of patients with upper GI cancer (10 events in the DOAC group and <5 events in the VKA group) and colorectal cancer (55 vs. 27 events, respectively), weighted bleeding rates were numerically higher in VKA users, consistent with non‐significantly lower HRs for GI bleeding in DOAC users: 0.61 (95% CI: 0.25, 1.52) for upper GI cancer and 0.92 (95% CI: 0.58, 1.46) for colorectal cancer (Table 3).

**TABLE 3:** One‐year rates and HRs for bleeding events in patients with atrial fibrillation and history of gastrointestinal cancer by subgroups according to initial prescription claim for a DOAC or VKA

Outcome	Group	Bleeding events, *n*	Unweighted rate per 100 person‐years (95% CI)	Weighted rate per 100 person‐years (95% CI)	Weighted HR (95% CI)
Active cancer					
Any bleeding	DOAC	55	11.92 (9.15, 15.52)	11.70 (8.97, 15.51)	1.12 (0.71, 1.76)
	VKA	25	9.03 (6.10, 13.36)	10.24 (6.90, 15.82)	Ref
GI bleeding	DOAC	27	5.66 (3.88, 8.26)	5.52 (3.81, 8.30)	1.00 (0.53, 1.88)
	VKA	14	5.00 (2.96, 8.45)	5.42 (3.17, 10.03)	Ref
Upper gastrointestinal cancer					
Any bleeding	DOAC	11	8.62 (4.77, 15.56)	8.79 (4.88, 17.41)	0.66 (0.27, 1.61)
	VKA	<5	6.74 (2.53, 17.95)	13.61 (2.38, 196.93)	Ref
GI bleeding	DOAC	10	7.83 (4.21, 14.56)	8.17 (4.40, 16.85)	0.61 (0.25, 1.52)
	VKA	<5	6.74 (2.53, 17.95)	13.61 (.38, 196.93)	Ref
Colorectal cancer					
Any bleeding	DOAC	115	10.65 (8.87, 12.78)	10.88 (9.06, 13.17)	0.94 (0.68, 1.29)
	VKA	52	10.78 (8.21, 14.14)	11.52 (8.74, 15.45)	Ref
GI bleeding	DOAC	55	4.95 (3.80, 6.45)	5.08 (3.92, 6.71)	0.92 (0.58, 1.46)
	VKA	27	5.47 (3.75, 7.97)	5.49 (3.76, 8.31)	Ref

Note

Counts were suppressed for observations with less than five incidents, to prevent disclosure of potentially identifiable information.

Abbreviations: DOAC, direct oral anticoagulant; VKA, vitamin K antagonist.

## 4 DISCUSSION

In this large, nationwide cohort study we observed similar 1‐year bleeding risks for DOAC compared with VKA among patients with AF and GI cancer. No clinically meaningful differences in bleeding risk were observed neither in GI cancer in general or in specific subgroups of upper GI or colorectal cancer. Our data, on the other hand, shows several important differences between patients receiving VKA versus DOAC.

Although AF patients with cancer in general are at increased risk of bleeding compared with non‐cancer patients, post‐hoc analyses from clinical trials demonstrated similar safety profiles for DOAC and VKA in cancer patients.^10^, ^13^, ^14^ These results were summarized in a meta‐analysis, which also included two cohort studies, and reported a lower risk of intracranial or GI bleeding as a composite endpoint with a relative risk of 0.65 (95% CI: 0.42, 0.98) for DOAC users.^26^ Similar results were reported in other cohort studies,^15^, ^16^, ^17^ but none of these focused on bleeding risk specifically for patients with GI cancer. A study from the United States including patients with and without cancer treated with anticoagulants for any indication, showed a bleeding risk in those treated with warfarin with a lumen GI cancer of 23.6% at six months after warfarin initiation, 20.4% for those treated with rivaroxaban, and 18.9% for patients initiating apixaban.^27^ A bleeding risk that was much higher than in our study, which followed patients with a history of cancer after initiation of anticoagulant treatment specifically for stroke prevention in patients with AF.^11^ We demonstrated a lower risk for intracranial hemorrhage associated with DOAC. Though we did not observe a strong association between DOAC and GI bleeding, our data demonstrated a tendency toward higher risks for patients with GI cancer initiating standard‐dose than reduced‐dose DOAC, which was similar in patients with active cancer.

The GI system may be prone to bleeding due to rich intra‐ and submucosal blood supply and high cell turnover. DOAC may have topical effects in the GI tract by direct inhibition of specific proteins involved in the coagulation cascade (factor IIa or factor Xa), whereas VKA have no direct anticoagulant effect. The P‐glycoprotein efflux pump regulate DOAC concentrations and mucosal exposure and may be overexpressed in some GI cancers.^28^ In our study, 22.4% of the DOAC users initiated treatment with dabigatran. The tartaric acid component of dabigatran may cause dyspepsia,^7^ and increase mucosal vulnerability. The absorption of dabigatran is very low (5%‐7%) and necessitates a high dose to ensure sufficient plasma dabigatran blood levels. Intraluminal activation of dabigatran etexilate is also hypothesized to increase bleeding risk.^29^ However, 40.9% of DOAC users were treated with apixaban, which does not seem to increase the risk of GI bleeding in cancer patients.^30^ Specifically, apixaban has been associated with a lower risk of major and GI bleeding compared with rivaroxaban and dabigatran.^31^ Differences in anticoagulant dosing, drug levels, and clearance may also affect bleeding risk, as well as concurrent treatment with aspirin, non‐steroidal anti‐inflammatory drugs, or other antiplatelet drugs. When we restricted to patients treated with standard‐dose DOAC, the HR estimates increased slightly for all bleeding endpoints except intracranial bleeding. Thus, the potential association with bleeding in our cohort is not explained by GI cancer patients at high baseline bleeding risk initiating reduced‐dose DOAC. For patients with active cancer, we also found a slightly elevated HR estimate, though imprecisely estimated, for the composite of all bleedings, but not for GI bleeding.

This study was based on a nationwide cohort of patients with AF and prevalent GI cancer who were new users of OAC and treated in a tax‐supported and uniformly organized health care system. All Danish hospitals report inpatient, outpatient, and emergency discharge diagnoses to the DNPR,^20^ and all outpatient pharmacy prescription claims are recorded in the DNPD.^21^ Cancers are most often histologically verified in the DCR, which is virtually complete and valid due to mandatory reporting throughout the Danish health care system.^22^ Our ability to identify patients in these registries, in a national setting with free access to health care, and track individuals by means of the CRS enabled unselected patient inclusion and complete follow‐up.^19^

### 4.1 Limitations

Due to the registry‐based design, we did not have information on use of low‐molecular‐weight‐heparin administered at hospitals, laboratory values, drug compliance, quality of VKA treatment, and lifestyle factors relevant for bleeding conditions. Choice of anticoagulant therapy may depend on patient and physician preference, cancer type, stage, and time since cancer diagnosis due to more difficult management in patients with active cancer, who are often hypercoagulable and treated with oncology drugs and radiotherapy or for concurrent conditions. Our comparative analysis; however, was based on weighted populations, which accounted for observed baseline imbalances between treatment groups, such as differences in prevalence of cardiovascular comorbidities and cancer types. We relied on hospital diagnoses without information on extent and severity of bleeding, and we could not determine whether some bleedings were limited to manageable bleedings with few complications. We did not consider treatments and incident conditions diagnosed during follow‐up, which may affect bleeding and mortality risk. The positive predictive values of the bleeding codes in the DNPR may vary according to bleeding site, extent, and severity.

## 5 CONCLUSION

In this nationwide cohort study of AF patients with a history of GI cancer, we observed no clinically meaningful difference in the 1‐year risk of bleeding or GI bleeding associated with DOAC compared with VKA. The findings were consistent in subgroups of patients with upper GI cancer or colorectal cancer. However, we did observe numerically higher HRs for the composite of all bleedings among patients with active cancer. The low number of bleeding events; however, precluded us from obtaining stable relative effectiveness estimates. Additional studies assessing bleeding risk for specific DOACs in AF patients with GI cancer are warranted.

## CONFLICT OF INTEREST

Dr. Søgaard reports personal fees from Bayer, outside the submitted work. Dr. Grove has received speaker honoraria or consultancy fees from AstraZeneca, Bayer, Boehringer Ingelheim, Bristol‐Myers Squibb, Pfizer, MSD, MundiPharma, Portola Pharmaceuticals, Lundbeck Pharma, and Roche. He is an investigator in the SATELLITE, ETESIAN, and FLAVOUR studies (AstraZeneca) and has received unrestricted research grants from Boehringer Ingelheim. Dr. Lip reports consultancy and speaker fees from BMS/Pfizer, Boehringer Ingelheim, and Daiichi‐Sanokyo outside the submitted work. No fees received personally. Dr. Larsen reports grants from Obel Family Foundation, personal fees from Bayer AG, personal fees from Pfizer, personal fees from Bristol Meyer Squibb, personal fees from MSD, personal fees from Bohringer Ingelheim, outside the submitted work. Dr. Nielsen reports personal fees from Bayer, personal fees and non‐financial support from Daiichi‐Sankyo, personal fees from BMS/Pfizer, outside the submitted work. The other authors have nothing to disclose.

## AUTHORS CONTRIBUTION

**Anne Ording**: Conceptualization, Methodology, Investigation, Formal analysis, Writing‐ Original draft preparation, Writing—Review & Editing, Project Administration. **Mette Søgaard**: Conceptualization, Methodology, Investigation, Writing‐ Original draft preparation, Writing—Review & Editing. **Flemming Skjøth**: Methodology, Investigation, Data Curation, Resources, Formal analysis, Software, Validation, Visualization, Writing‐ Original draft preparation, Writing—Review & Editing. **Erik Grove**: Conceptualization, Writing – Review & Editing. **Gregory Lip**: Conceptualization, Writing—Review & Editing. **Torben Larsen**: Conceptualization, Methodology, Investigation, Funding acquisition, Resources, Writing‐ Original draft preparation, Writing—Review & Editing. **Peter Nielsen**: Methodology, Investigation, Supervision, Data Curation, Resources, Formal analysis, Software, Validation, Visualization, Writing‐ Original draft preparation, Writing—Review & Editing.

## ETHICS

The study was conducted in compliance with the General Data Protection Regulation Article 30, recorded at Aalborg University Hospital and Aalborg University (record no: 2017‐509‐00006). Danish law does not require ethical approval or informed consent from patients in studies based on routinely collected registry data.

## ROLE OF THE FUNDING SOURCE

The funding source had no role in study design; in the collection, analysis, and interpretation of data; in the writing of the report; and in the decision to submit the paper for publication. All authors confirm that they had full access to all the data in the study and accept responsibility to submit for publication.

## Supporting information

Supplementary Material

## Data Availability

Our own approvals to use the data sources for the current study do not allow us to distribute or make patient data directly available to other parties. Interested researchers may apply for data access through the Research Service at the Danish Health Data Authority (e‐mail: forskerservice@sundhedsdata.dk; phone: +45 3268 5116). Up‐to‐date information on data access is available online (http://sundhedsdatastyrelsen.dk/da/forskerservice). Access to data from the Danish Health Data Authority requires approval from the Danish Data Protection Agency (https://www.datatilsynet.dk/english/the‐danish‐data‐protection‐agency/introduction‐to‐the‐danish‐data‐protection‐agency/).
