# Supplementary material for: Bleeding complications in patients with gastrointestinal cancer and atrial fibrillation treated with oral anticoagulants
Source: Cancer Med. 2021 Jun 11;10(13):4405–14. doi: 10.1002/cam4.4012 (PMC8267127; doi:10.1002/cam4.4012)

**SUPPORTING INFORMATION**

**SUPPORTING INFORMATION TABLE 1.** Diagnostic codes and definitions.

| **VARIABLE** | **INTERNAL CLASSIFICATION OF DISEASE, 10^TH^ REVISION** | **ANATOMIC THERAPEUTIC CHEMICAL CLASSIFICATION** |
| --- | --- | --- |
| Atrial Fibrillation | I48 |  |
| Esophageal cancer | C15 |  |
| Stomach cancer | C16 |  |
| Small intestine | C17 |  |
| Colon cancer | C18 C19 |  |
| Rectal cancer | C20 |  |
| Anal cancer | C21 |  |
| Liver and gall bladder cancer | C22 C23 C24 |  |
| Pancreatic cancer | C25 |  |
| Stroke | I63 I64 |  |
| Systemic Embolism | I74 |  |
| Myocardial infarction | I21 I23 |  |
| Ischemic heart disease | I20 I21 I23 I24 I25 |  |
| Congestive Heart Failure | I110 I130 I132 I420 I50 |  |
| Valvular disease | I05 I06 I07 I080 I098 I34 I35 I36 I37 I390 I393 I511A Q22 |  |
| Cardiomyopathy | I42 I43 |  |
| Venous thromboembolism | I26 I801 I802 I803 I808 I809  I819 I636 I676 I822 I823 I828  I829 |  |
| Obesity | E65 E66 E67 E68 |  |
| Hyperthyroidism | E05 E06 |  |
| Chronic Pulmonary Disease | J40 J41 J42 J43 J44 J45 J46  J47 J60 J61 J62 J63 J64 J65  J67 J684 J701 J703 J841 J920  J921 J982 J983 |  |
| Moderate/Severe Liver Disease | B150 B160 B162 B190 K704  K72 K766 I85 |  |
| Moderate/severe renal disease | I12 I13 N00 N01 N02 N03 N04  N05 N07 N11 N14 N17 N18  N19 Q61 |  |
| Intracranial hemorrhage | I60 I61 I62 I690 I691 I692 |  |
| Gastrointestinal bleeding | K250 K252 K254 K260 K262 K264 K270 K272 K274 K280  K282 K290 K921 K922 I850  I864A K228F K284 K298A  K625 K638B K638C K661  K838F K868G |  |
| Major bleeding | D62 J492 H356 H431 N02  R04 R31 G951A H052A H313  H450 I312 |  |
| Thrombophlepitis | I800A |  |
| Gastro intestinal surgery | KJ |  |
| Chemotherapy | BWHA |  |
| Radiotherapy | BWGC BWGE BWGG BWGJ BNGE BNGF KAAG50 BAG BG BCG BDGBEG BGG BHG BIG BLG BJG BOG |  |
| Warfarin |  | B01AA03 |
| Phenprocoumon |  | B01AE07 |
| Apixaban |  | B01AF02 |
| Dabigatran |  | B01AE07 |
| Rivaroxoban |  | B01AF01 |
| Edoxaban |  | B01AF03 |
| Diuretics |  | C03 |
| Renin-angiotensin inhibitor (ARB or ACE  inhibitor) |  | C09 |
| Calcium channel blocker |  | C07F C08 C09BB C09DB |
| Beta blocker |  | C07 |
| Digoxin |  | C01AA05 |
| Lipid lowering drugs |  | C10 |
| Aspirin |  | B01AC06 |
| Non-steroidal anti-inflammatory drug |  | M01AA M01AB M01AC M01AE M01AG M01AH M01AX01 |
| Amiodarone |  | C01BD01 |
| Clopidogrel, ticagrelor,  prasugrel |  | B01AC04 B01AC24 B01AC22 |
| Congestive heart failure | I110 I130 I132 I420 I50 | C03C |
| Left ventricular dysfunction | I501 I509 |  |
| Diabetes Mellitus | E100 E101 E109 E110 E111 E119 | A10 |
| Transient ischemic disease | G45 |  |
| Systemic Embolism | I74 |  |
| Myocardial infarction | I21 I23 |  |
| Peripheral vascular/ischemic disease | I702 I703 I704 I705 I706 I707 I708 I709 I71  I739 |  |
| Aortic Plaque | I700 |  |
| Alfa adrenic block |  | C02A C02B C02C |
| Non-loop diuretics |  | C02DA C02L C03A C03B C03D C03EA  C03X C07C C07D C09BA C09DA  C09XA52 |
| Vasodilator |  | C02DB C02DD C02DG C04 C05 |
| Beta blocker |  | C07 |
| Calcium channel blocker |  |  |

**SUPPORTING INFORMATION TABLE 2.** Characteristics of patients with atrial fibrillation and a history of gastrointestinal cancer according to initial prescription claim for a reduced-dose or standard-dose DOAC.

|  | **REDUCED-DOSE DOAC** | **STANDARD-DOSE DOAC** |
| --- | --- | --- |
| Participants | 609 | 867 |
| Female | 55.8 (340) | 36.3 (315) |
| Median age, y | 84.0 (79.0-88.0) | 75.0 (70.0-80.0) |
| Cancer type* |  |  |
| Pancreatic cancer | 1.8 (11) | 1.8 (16) |
| Liver cancer | 1.6 (10) | 2.3 (20) |
| Esophagus cancer | 2.3 (14) | 3.1 (27) |
| Stomach cancer | 4.4 (27) | 5.5 (48) |
| Small intestinal cancer | - (<5) | 1.7 (15) |
| Colorectal | 88.0 (536) | 85.5 (741) |
| Anal canal | 1.6 (10) | 0.8 (7) |
| Metastasis | 1.6 (10) | 2.5 (22) |
| Active cancer | 37.3 (227) | 42.0 (364) |
| Cancer treatment** |  |  |
| Chemotherapy | 6.6 (40) | 9.2 (80) |
| Radiation therapy | 28.7 (175) | 31.4 (272) |
| Surgery | 17.1 (104) | 24.5 (212) |
| Cancer stage |  |  |
| Localized | 53.4 (325) | 46.7 (405) |
| Regional | 20.2 (123) | 27.8 (241) |
| Distant | 4.9 (30) | 4.7 (41) |
| Unknown | 21.5 (131) | 20.8 (180) |
| Comorbidities |  |  |
| Heart failure | 29.2 (178) | 19.1 (166) |
| Diabetes | 16.7 (102) | 17.4 (151) |
| Hypertension | 61.4 (374) | 55.0 (477) |
| Stroke | 17.1 (104) | 12.8 (111) |
| Systemic embolism | - (<5) | 0.9 (8) |
| Myocardial infarction | 12.5 (76) | 8.2 (71) |
| Ischemic heart disease | 26.1 (159) | 22.0 (191) |
| Cardiomyopathy | 2.0 (12) | 1.6 (14) |
| Obesity | 7.1 (43) | 9.5 (82) |
| Hyperthyroidism | 3.9 (24) | 3.7 (32) |
| Chronic pulmonary disease | 18.1 (110) | 13.4 (116) |
| Liver disease | - (<5) | - (<5) |
| Renal disease | 8.9 (54) | 3.9 (34) |
| Previous bleeding | 29.2 (178) | 20.4 (177) |
| HAS-BLED score |  |  |
| 0 | 0.8 (5) | 1.2 (10) |
| 1-2 | 41.7 (254) | 52.4 (454) |
| 3+ | 57.5 (350) | 46.5 (403) |
| CHA_2_DS_2_-VASc score |  |  |
| 0 | - (<5) | 1.3 (11) |
| 1 | - (<5) | 11.2 (97) |
| 2-4 | - (<5) | 68.5 (594) |
| 5+ | - (<5) | 19.0 (165) |
| Medication |  |  |
| Apixaban | 44.7 (272) | 38.2 (331) |
| Dabigatran | 30.7 (187) | 16.6 (144) |
| Edoxaban | - (<5) | 1.2 (10) |
| Rivaroxaban | 24.0 (146) | 44.1 (382) |
| Renin-angiotensin inhibitor (ACE/ARB) | 39.2 (239) | 34.1 (296) |
| Calcium channel blockers | 21.8 (133) | 19.4 (168) |
| Beta blockers | 61.1 (372) | 62.3 (540) |
| Diuretics | 42.9 (261) | 28.6 (248) |
| Digoxin | 28.1 (171) | 20.5 (178) |
| Lipid lowering drugs | 27.4 (167) | 29.0 (251) |
| Aspirin | 29.4 (179) | 22.1 (192) |
| NSAID | 5.3 (32) | 8.1 (70) |
| Amiodarone | 3.4 (21) | 3.3 (29) |
| Thienpyridines (clopidogrel, ticagrelor, prasugrel) | 11.7 (71) | 8.3 (72) |

Data are the median (interquartile range) or the % (number of patients), as indicated; counts were suppressed for observations with less than five incidents, to prevent disclosure of potentially identifiable information; *cancer types are not mutually exclusive; **recorded within six months before index; DOAC: direct oral anticoagulant.

**SUPPORTING INFORMATION FIGURE 1.** Distribution of the propensity score according to treatment.


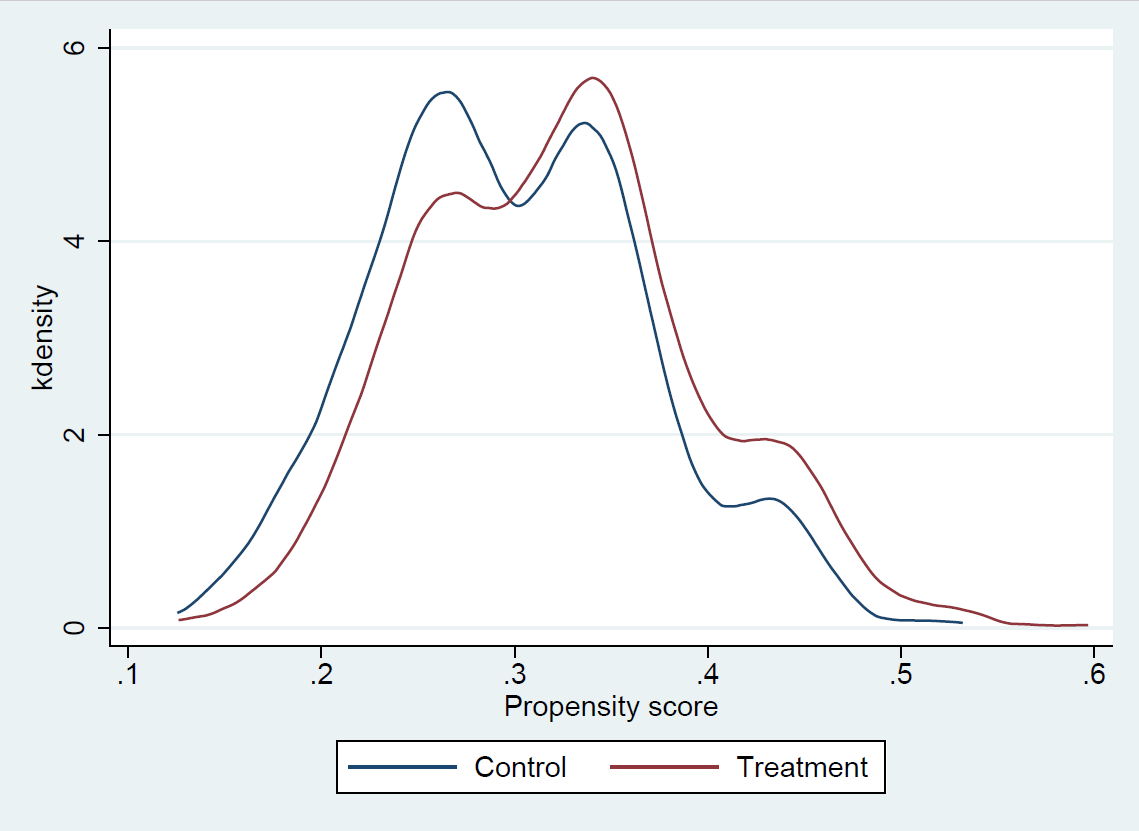

Supplement: Supplementary file 1 — Supplementary Material [file CAM4-10-4405-s001.docx]
